# Vision facilitates tactile perception when grasping an object

**DOI:** 10.1038/s41598-018-33916-8

**Published:** 2018-10-23

**Authors:** Georgiana Juravle, Francisco L. Colino, Xhino Meleqi, Gordon Binsted, Alessandro Farnè

**Affiliations:** 1 Integrative Multisensory Perception Action & Cognition Team - ImpAct, Lyon Neuroscience Research Center, INSERM U1028, CNRS U5292 Lyon, France; 2 University of Lyon 1, Lyon, France; 3 Centre for Biomedical Research, University of Victoria, Victoria, Canada; 4 Faculty of Health and Social Development, School of Health and Exercise Sciences, University of British Columbia, Kelowna, Canada; 5 Hospices Civils de Lyon, Mouvement & Handicap, Neuro-immersion, Lyon, France

## Abstract

Tactile sensitivity measured on the hand is significantly decreased for a moving (MH), as opposed to a resting hand (RH). This process (i.e., tactile suppression) is affected by the availability of visual information during goal-directed action. However, the timing of the contribution of visual information is currently unclear for reach-to-grasp movements, especially in the period before the digits land on the object to grasp it. Here participants reached for, grasped, and lifted an object placed in front of them in conditions of full/limited vision. Tactile perception was assessed by measures of signal detection theory (*d’* & *c’*). Electro-cutaneous stimulation could be delivered/not at the MH/RH, either during movement preparation, execution, before grasping, or while lifting the object. Results confirm tactile gating at the MH. This result is accompanied by a significant conservative criterion shift at the MH for the latter movement stages. Importantly, visual information enhances MH sensitivity just before grasping the object, but also improves RH sensitivity, during object lift. These findings reveal that tactile suppression is shaped by visual inputs at critical action stages. Further, they indicate that such a time-dependent modulation from vision to touch extends beyond the MH, suggesting a dynamic monitoring of the grasp space.

## Introduction

Tactile suppression is a well-known phenomenon characterized by a decrement in tactile sensitivity, typically occurring on our upper limbs in relation to movements that we perform. Also known as tactile attenuation, or simply as gating, tactile suppression has been found in a multitude of motor tasks, by utilizing a wide array of tactile sensitivity measurements (see^1^, for a review). This study focuses on the sensory suppression known to occur in goal-directed reach-to-grasp movements. Our aim is to test whether and how vision modulates tactile gating manifestation.

Tactile suppression is closely intertwined with movement, with the *timing of tactile stimulation* being the first determining factor of tactile suppression^2^. For example, in an earlier study, participants were asked to make repeated reach-to-grasp movements for an object placed in front of them, in line with a series of auditory tones. A discrimination task was used to measure tactile sensitivity. Specifically, participants decided which one of two stimuli delivered to their resting left hand and their moving right hand was stronger, with stimulation delivered at various times during the movement, from preparation, through execution, and post-movement phases. Results indicated tactile suppression, that is, higher thresholds (or poorer performance) during movement execution, as compared to both preparation and post-movement phases, with no significant difference in sensitivity between these two^3^, see also^4,5^, for a replication. Tactile suppression typically makes an appearance during goal-directed movement and it has comparable profiles for either the right or the left hand moving.

The next factor determining gating in the tactile domain is *context-dependence*. Contextual influences on suppression are differently approached by different labs working on tactile suppression: That is, suppression has been shown to be highly-dependent on the exact body part involved in the movement or not (i.e., relevance in tactile suppression^6–8^). Further, tactile suppression has been shown to be highly affected by the motor task at hand (e.g., active versus passive reaches, exploratory movements versus reaches^9^; see also pantomimed movements^5^; as well as precision reaching^10^). Lastly, and perhaps the factor with the largest influence, is the exact *type of tactile task*, or the specific dependent measure used to assess tactile suppression in relation to movement. Most likely owing to the tradition in visual science, the majority of tactile suppression studies have focused on measuring tactile thresholds to assess suppression. Extensive psychophysics is fundamental for understanding the tactile suppression phenomenon, but this approach comes at the cost of having threshold measures hard to directly compare across labs (e.g., how to *easily* compare thresholds provided in milliampère to those in decibels) and, most importantly, thresholding alone cannot account for criterion changes in the data. Yet, criterion shifts appear to consistently contribute to tactile suppression (i.e., not only do participants feel less when they move, they are also less inclined to report the presence of a tactile stimulus), therefore, tactile suppression needs to *always* be assessed with appropriate measures of response bias^1^.

Here, we focus on the relevance aspect of tactile suppression, by delivering touches at the index finger involved in the grasp at different timings during movement. Our starting point is the crucial finding that tactile suppression manifests differently at each digit involved in the process of reaching and grasping an object. Colino and his colleagues were the first to demonstrate that the index finger involved in a grasping action experiences less suppression, as compared to the little finger not participating in the grasp, or the completely unrelated forearm of the resting hand^7,11^. Further studies have attempted to replicate and extend claims on this finding; however, their methods violated the first rule of the timing of tactile suppression, by having delivered stimulation either too early (i.e., at movement initiation when suppression is maximal^12^), or too late (i.e., once the movement has terminated^5^). Having convincingly established the relevance of the motor effector when assessing tactile suppression, the authors next investigated whether the tactile suppression effect is affected by the availability of visual information during movement. For this, they had their participants perform reach-to-grasp movements under conditions of full vision, or limited visual availability, with only a short period of fixation at the beginning of the movement, and the rest of the movement performed with vision occluded. Their results indicated that visual information availability contributes to decrease the overall magnitude of tactile suppression experienced during movement^6^.

To assess the temporal profile of vision’s contribution to tactile suppression, here we consider the tactile stimulation delivery timing, the effector relevance, and the requirements for tactile perception measurement during movement. For this, we define timing based on real-time spatial coordinates of the hand, as opposed to stimulation delivery relative to the imperative cue, as it was previously studied as far as relevance in tactile suppression is concerned^5,7,11^. Our participants reached for and grasped an object placed in front of them, under conditions of full visual information or limited visual information. Because we were interested in the timing of contact with the object (i.e., to investigate tactile facilitation given by any feedback from the tactile receptors involved in the grasp), we defined the different timings *spatially*. That is, we utilized the traditional timings of preparation and execution, but also added two timings for tactile stimulus delivery: *(1*) the ‘just before grasp’ timing, where the index and thumb are within less than half a centimetre from landing on the goal object and, *(2)* the ‘while lifting’ timing, when the digits have landed on the goal object, and they are now immobile, but they are nevertheless engaged in holding it and lifting it off the table surface. Tactile stimulation could be delivered, with equal probability, to either the moving or the resting hand. To assess criterion change, 50% of trials had no tactile stimulus delivered, thus, all the behavioural results reported are based on signal detection theory measures such as sensitivity (*d’*) and the relative criterion location, denoted as *c’*^13,14^.

We hypothesized that any (sensory feedback-driven) contribution to tactile sensitivity just before grasping the object and/or lifting it should be evident in a significantly improved tactile performance measured at the moving hand, as opposed to performance at the resting hand. Additionally, if visual information were to be responsible for what is felt as the hand lands on an object of interest (i.e., in connection to the well-researched visual preference for the index finger in reach-to-grasp tasks^15,16^), then we expect a significantly better moving hand sensitivity in those conditions where vision is available during reach, as opposed to the reaches performed under limited visual information.

## Results

### Behaviour

**Figure 1:**
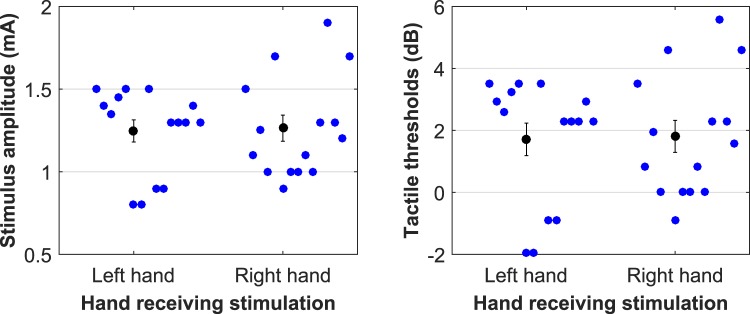
Scatter plots on individual threshold data recorded at rest (in blue) together with their mean (in black), plotted in both mA (left panel), as well as a ratio (dB, right panel). Vertical error bars represent the standard error of the mean.

**Figure 2:**
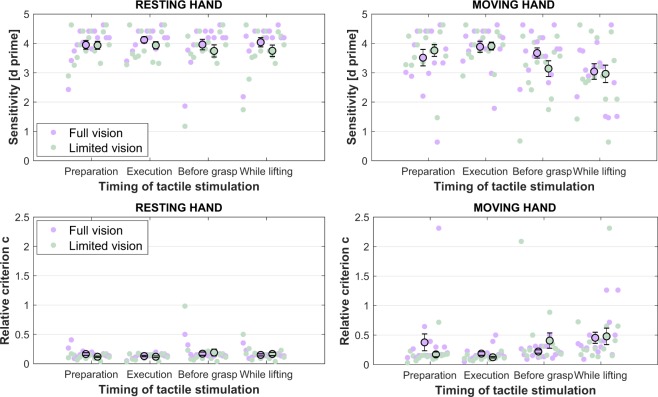
Scatter plots of individual sensitivity (*d’*) data (upper row) and relative criterion *c’* data (lower row) together with means and their corresponding standard error.

Mean tactile detection thresholds derived at rest are presented in Fig. 1. No significant difference was recorded between participants’ left hand detection threshold and participants’ right hand detection threshold at rest [*t*(14) = 0.22, *p* = 0.832, *r* = 0.452]. Importantly no false alarms were detected in the thresholding procedure for our sample of 15 participants. Scatter plots of individual sensitivity data together with their corresponding means are presented in Fig. 2. Means with standard error for the two dependent measures collected are presented in Table 1.

**Table 1:** Mean behavioural data (*d*’ and *c*’ over rows) for all conditions tested.

	Preparation				Execution				Before grasp				While lifting			
	Full vision		Limited vision		Full vision		Limited vision		Full vision		Limited vision		Full vision		Limited vision	
	Rest	Move	Rest	Move	Rest	Move	Rest	Move	Rest	Move	Rest	Move	Rest	Move	Rest	Move
d’	3.9 (0.6)	3.5 (0.3)	3.9 (0.5)	3.8 (0.2)	4.1 (0.4)	3.9 (0.2)	3.9 (0.5)	3.9 (0.1)	4.0 (0.7)	3.7 (0.2)	3.7 (0.8)	3.1 (0.3)	4.0 (0.6)	3.0 (0.3)	3.7 (0.7)	3.0 (0.3)
c’	0.17(0.02)	0.38 (0.1)	0.12 (0.01)	0.17 (0.04)	0.13 (0.01)	0.19 (0.03)	0.12 (0.01)	0.12 (0.01)	0.17 (0.03)	0.2 (0.03)	0.19 (0.05)	0.41 (0.13)	0.15 (0.02)	0.45 (0.09)	0.17 (0.03)	0.48 (0.14)

### Sensitivity (d’)

The existence of sensory suppression was clearly indicated with a significant main effect of TIMING [*F*(3,42) = 8.84, *p* < 0.001, *η*^2^_*p*_ = 0.387]. That is, tactile sensitivity was significantly lower while lifting the object [*M* = 3.45, *SE* = 0.20] as compared to while preparing the movement [*M* = 3.79, *SE* = 0.16, *F*(1,14) = 9.02, *p* = 0.009, *η*^2^_*p*_ = 0.392]; Similarly, significant perceptual decrements were evident for the preparatory phase [*F*(1,14) = 6.82, *p* = 0.021, *η*^2^_*p*_ = 0.327], the just before grasp phase [*M* = 3.63, *SE* = 0.19, *F*(1,14) = 9.62, *p* = 0.008, *η*^2^_*p*_ = 0.407], and the lifting phase [*F*(1,14) = 26.57, *p* < 0.001, *η*^2^_*p*_ = 0.655], in relation to the execution phase [*M* = 3.96, *SE* = 0.12]. A significant main effect of HAND [*F*(1,14) = 14.35, *p* = 0.002, *η*^2^_*p*_ = 0.506] indicated that the resting hand sensitivity [*M* = 3.93, *SE* = 0.14] was, as expected, significantly higher than that of the moving hand [*M* = 3.49, *SE* = 0.19].

A significant two-way interaction between TIMING and VISION AVAILABILITY [*F*(3,42) = 5.76, *p* = 0.002, *η*^2^_*p*_ = 0.292] was found; post hoc tests indicated that this was given by participants being significantly more sensitive to tactile stimulation in the before grasp timing under conditions of full vision [*M* = 3.82, *SE* = 0.16], as compared to the same timing, but when no vision was available [*M* = 3.44, *SE* = 0.23, *t*(14) = 3.06, *p* = 0.008, *r* = 0.861]. Furthermore, a significant interaction between TIMING and HAND was also found on the *d* prime data [*F*(3,42) = 7.85, *p* < 0.001, *η*^2^_*p*_ = 0.359]. Participants were significantly more sensitive to detect the tactile stimulus at the resting hand for both the before grasp [*M* = 3.85, *SE* = 0.19], as well as the while lifting the object conditions [*M* = 3.89, *SE* = 0.17], as compared to their moving hand performance for the same timings of the movement [before grasp: *M* = 3.41, *SE* = 0.20, *t*(14) = 3.52, *p* = 0.003, *r* = 0.795; while lifting: *M* = 3.00, *SE* = 0.27, *t*(14) = 4.24, *p* < 0.001, *r* = 0.628].

Lastly, a three-way interaction between TIMING, VISION AVAILABILITY, and HAND proved to be significant [*F*(3,42) = 3.20, *p* = 0.033, *η*^2^_*p*_ = 0.186]. In accordance with our hypothesis, we looked at the significant two-way interactions, for each of the resting and the moving hands.

For the resting hand, the main effects of TIMING [*F*(3,42) = 1.59, *p* = 0.207, *η*^2^_*p*_ = 0.102] and VISION AVAILABILITY [*F*(1,14) = 3.71, *p* = 0.075, *η*^2^_*p*_ = 0.209] failed to reach statistical significance. The interaction between the two factors, at the limit of significance [*F*(3,42) = 2.82, *p* = 0.050, *η*^2^_*p*_ = 0.168] was given by participants’ sensitivity being higher for the full vision condition [*M* = 4.04, *SE* = 0.16], as compared to the limited vision condition [*M* = 3.75, *SE* = 0.19], only when participants were lifting the object [*t*(14) = 2.91, *p* = 0.011, *r* = 0.858].

In what regards the moving hand, no main effect of VISION AVAILABILITY was found [*F*(1,14) = 0.622, *p* = 0.443, *η*^2^_*p*_ = 0.043], but a significant main effect of TIMING [*F*(3,42) = 10.21, *p* < 0.001, *η*^2^_*p*_ = 0.422, *ε = *0.706]. Planned comparisons indicated a significant performance drop while lifting the object [*M* = 3.00, *SE* = 0.27] as compared to both preparing the movement [*M* = 3.64, *SE* = 0.23, *F*(1,14) = 11.02, *p* = 0.005, *η*^2^_*p*_ = 0.440], and to before grasping the object [*M* = 3.41, *SE* = 0.20, *F*(1,14) = 5.28, *p* = 0.038, *η*^2^_*p*_ = 0.274]. Further, participants’ sensitivity was significantly lower in the preparation [*F*(1,14) = 5.24, *p* = 0.038, *η*^2^_*p*_ = 0.272], before grasp [*F*(1,14) = 15.55, *p* = 0.001, *η*^2^_*p*_ = 0.526], and while lifting periods [*F*(1,14) = 28.67, *p* < 0.001, *η*^2^_*p*_ = 0.672], as compared to the execution period [*M* = 3.90, *SE* = 0.15].

Lastly, a significant two-way interaction between TIMING and VISION AVAILABILITY [*F*(3,42) = 5.04, *p* = 0.004, *η*^2^_*p*_ = 0.265] was evident in the moving hand *d’* data. Post hoc tests indicated that this effect was stemming from the measured moving right hand sensitivity in the full vision condition [*M* = 3.67, *SE* = 0.17] being significantly higher as compared to the limited vision condition [*M* = 3.14, *SE* = 0.27], specifically in the timing of just before grasping the goal object [*t*(14) = 2.78, *p* = 0.014, *r* = 0.703].

### Relative criterion c’

We concentrate our discussion of the criterion results strictly on those reflecting the relative criterion *c’*, i.e., the criterion location *c* scaled by sensitivity. It is advised that for those studies where *d’* differs between experimental conditions (such is the case of the present report), sensitivity to be taken into account when considering and discussing response bias^14^.

The analysis indicated a significant main effect of HAND [*F*(1,14) = 7.17, *p* = 0.018, *η*^2^_*p*_ = 0.339], with participants more likely to say no tactile stimulus was present when stimulation was delivered at their moving hand [*M* = 0.30, *SE* = 0.05], as compared to when stimulation was delivered to their resting hand [*M* = 0.15, *SE* = 0.02]. In addition, a significant main effect of TIMING was found [*F*(3,42) = 3.39, *p* = 0.027, *η*^2^_*p*_ = 0.195, *ε = *0.655]. Planned comparisons indicated that this was given by participants’ criterion in the lifting timing of the movement being significantly more conservative [*M* = 0.31, *SE* = 0.05], as compared to both the preparation [*M* = 0.21, *SE* = 0.04, *F*(1,14) = 6.73, *p* = 0.021, *η*^2^_*p*_ = 0.325], and execution periods of the movement [*M* = 0.14, *SE* = 0.01, *F*(1,14) = 12.88, *p* = 0.003, *η*^2^_*p*_ = 0.479].

**Figure 3:**
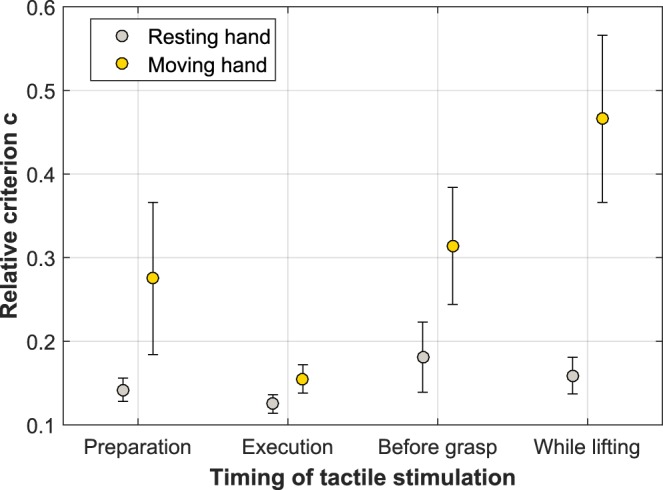
Depiction of the timing by hand interaction on the average relative criterion *c’* data. With 0 taken to reflect a point of no bias, positive values of relative criterion *c’* indicate a general inclination to respond ‘NO’. Vertical error bars represent the standard error of the mean.

Furthermore, a significant two-way interaction between TIMING and HAND was identified for the relative criterion *c’* data [*F*(3,42) = 3.72, *p* = 0.046, *η*^2^_*p*_ = 0.210, *ε = *0.566]. Post hoc tests indicated that for the before grasp period, participants were clearly more inclined to report that no stimulus was presented for the before grasp period when stimulation was delivered at the moving hand [*M* = 0.31, *SE* = 0.07], as compared to the resting hand [*M* = 0.18, *SE* = 0.04, *t*(14) = 3.19, *p* = 0.006, *r* = 0.841]. Similarly, participants were significantly more conservative in reporting moving hand stimuli once the reach was concluded and they were lifting the object [*M* = 0.46, *SE* = 0.10], as compared to stimuli delivered to the resting hand for the same lifting timing [*M* = 0.16, *SE* = 0.02, *t*(14) = 2.87, *p* = 0.012, *r* = −0.224]; see Fig. 3.

### Movement kinematics

Means together with their standard error for all the dependent measures considered for analysis are presented in Table 2. Due to the extensive amount of data analysed, we only report those main effects and interactions that were found to be significant in the present study.

**Table 2:** Mean kinematic data together with SEs. RTs – reaction times, MT – total movement time, PGA – peak grip aperture, TPGA – time to peak grip aperture, PV – peak velocity, TPV – time to peak velocity, PA – peak acceleration, TPA – time to peak acceleration, PD – peak deceleration, TPD – time to peak deceleration.

	Preparation				Execution				Before grasp				While lifting			
	Full vision		Limited vision		Full vision		Limited vision		Full vision		Limited vision		Full vision		Limited vision	
	Rest	Move	Rest	Move	Rest	Move	Rest	Move	Rest	Move	Rest	Move	Rest	Move	Rest	Move
RTs, ms	721 (29)	713 (33)	601 (23)	610 (30)	766 (34)	774 (35)	670 (32)	673 (33)	787 (35)	810 (40)	666 (29)	701 (26)	773 (34)	783 (38)	707 (41)	672 (27)
MT, ms	875 (39)	877 (40)	926 (29)	926 (34)	882 (42)	881 (40)	927 (35)	950 (35)	891 (42)	877 (38)	943 (38)	928 (34)	879 (39)	886 (42)	941 (43)	931 (39)
PGA, mm	162 (4)	161 (4)	188 (5)	189 (5)	161 (4)	160 (4)	187 (5)	187 (5)	162 (4)	161 (4)	189 (5)	188 (5)	161 (4)	161 (5)	187 (5)	188 (4)
TPGA, ms	695 (11)	697 (11)	769 (9)	767 (8)	695 (12)	690 (11)	770 (9)	776 (10)	700 (11)	702 (12)	772 (8)	771 (12)	686 (10)	694 (10)	784 (9)	763 (10)
PV, m/s	1.4 (0.06)	1.4 (0.06)	1.3 (0.06)	1.3 (0.05)	1.4 (0.06)	1.4 (0.06)	1.3 (0.6)	1.3 (0.06)	1.4 (0.06)	1.4 (0.06)	1.3 (0.06)	1.3 (0.06)	1.4 (0.06)	1.4 (0.06)	1.3 (0.06)	1.3 (0.06)
TPV, ms	405 (17)	401 (19)	396 (15)	396 (17)	403 (18)	406 (18)	406 (17)	409 (18)	412 (20)	400 (18)	412 (19)	398 (16)	396 (17)	407 (21)	410 (20)	405 (20)
PA, m/s^2^	6.5 (0.4)	6.5 (0.4)	6.2 (0.4)	6.3 (0.4)	6.2 (0.4)	6.3 (0.4)	6.0 (0.4)	6.0 (0.4)	6.2 (0.4)	6.3 (0.3)	5.9 (0.4)	6.1 (0.4)	6.3 (0.4)	6.3 (0.4)	6.0 (0.4)	6.1 (0.4)
TPA, ms	206 (19)	207 (21)	205 (20)	201 (17)	205 (19)	208 (18)	208 (18)	211 (20)	217 (20)	209 (19)	213 (21)	210 (15)	205 (19)	209 (19)	215 (18)	205 (18)
PD, m/s^2^	5.5 (0.4)	5.3 (0.4)	5.1 (0.4)	5.0 (0.3)	5.3 (0.4)	5.3 (0.4)	4.9 (0.3)	5.0 (0.4)	5.3 (0.4)	5.3 (0.4)	4.9 (0.4)	5.0 (0.4)	5.4 (0.4)	5.4 (0.5)	4.8 (0.4)	5.0 (0.4)
TPD, ms	576 (23)	580 (24)	555 (19)	558 (20)	576 (24)	574 (21)	564 (20)	566 (24)	589 (25)	576 (24)	575 (22)	558 (21)	579 (26)	578 (27)	582 (27)	566 (24)

### Timing of tactile stimulation

Reaction times differed as a function of the TIMING of tactile stimulation delivery [*F*(1,13) = 25.47, *p* < 0.001, *η*^2^_*p*_ = 0.662]. Specifically, participants’ RTs were significantly faster in the preparation period [*M* = 660.97 ms, *SE* = 25.83 ms], as compared to execution [*M* = 720.55 ms, *SE* = 30.90 ms, *F*(1,13) = 37.17, *p* < 0.001, *η*^2^_*p*_ = 0.741], before grasp [*M* = 740.68 ms, *SE* = 30.46 ms, *F*(1,13) = 46.04, *p* < 0.001, *η*^2^_*p*_ = 0.780], and while lifting periods [*M* = 733.83 ms, *SE* = 30.81 ms, *F*(1,13) = 21.87, *p* < 0.001, *η*^2^_*p*_ = 0.627], with the execution period RTs significantly faster than the before grasp period [*F*(1,13) = 7.15, *p* = 0.019, *η*^2^_*p*_ = 0.355]. Further, a main effect of TIMING of tactile stimulation delivery was found on the mean peak grip aperture [PGA, *F*(3,39) = 3.59, *p* = 0.022, *η*^2^_*p*_ = 0.216]. Planned comparisons indicated that the average PGA was significantly smaller when stimulation was delivered during the execution period of the movement [*M* = 173.68 mm, *SE* = 4.03 mm], as compared to both the preparatory phase [*M* = 175.06 mm, *SE* = 4.32 mm, *F*(1,13) = 5.15, *p* = 0.041, *η*^2^_*p*_ = 0.284], and the just before grasp phase [*M* = 175.05 mm, *SE* = 4.34 mm, *F*(1,13) = 6.57, *p* = 0.024, *η*^2^_*p*_ = 0.336]. Similarly, the mean peak acceleration (PA) was also influenced by the TIMING of tactile stimulation delivery [*F*(3,39) = 3.68, *p* = 0.020, *η*^2^_*p*_ = 0.220]. This effect was given by the participants’ reaches exhibiting on average a significantly elevated PA for those times when the tactile stimulation was delivered during the preparatory phase of the movement [*M* = 6.36 m/s, *SE* = 0.34 m/s], as compared to the execution period [*M* = 6.14 m/s, *SE* = 0.39 m/s, *F*(1,13) = 9.16, *p* = 0.010, *η*^2^_*p*_ = 0.413], the just before grasp period [*M* = 6.12 m/s, *SE* = 0.37 m/s, *F*(1,13) = 5.33, *p* = 0.038, *η*^2^_*p*_ = 0.295], as well as the lifting period of the movement [*M* = 6.18 m/s, *SE* = 0.40 m/s, *F*(1,13) = 8.95, *p* = 0.010, *η*^2^_*p*_ = 0.408]. The higher PA for stimulation delivered in the preparatory phase, together with the reaction times found to be faster for the same period could signal the typical arousal effect found for reaction times, which are faster when a tactile stimulus is delivered in connection to another sensory stimulus, the go signal to initiate the movement in our case^17,18^.

### Vision availability

**Figure 4:**
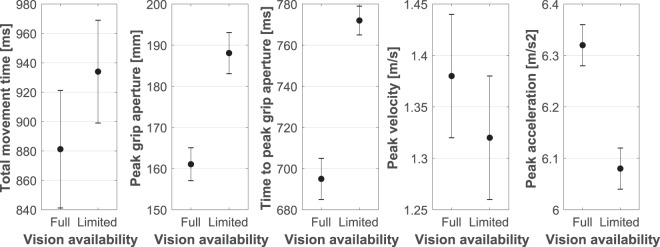
Depiction of the vision availability main effect for various kinematic markers tested (from left to right: total movement time, peak grip aperture, time to peak grip aperture, peak velocity, and peak acceleration). Vertical error bars represent the standard error of the mean.

Availability of vision affected most kinematic measures throughout the duration of the reach-to-grasp movement, see Fig. 4. That is, participants were significantly slower to initiate the movement under conditions of full vision [*M* = 766 ms, *SE* = 33 ms] as compared to the limited vision movement condition [*M* = 662 ms, *SE* = 26 ms, *F*(1,13) = 47.15, *p* < 0.001, *η*^2^_*p*_ = 0.784]. Their total movement time was significantly longer under conditions of limited vision [*M* = 934 ms, *SE* = 35 ms] as compared to the full vision movement condition [*M* = 881.21 ms, *SE* = 40 ms, *F*(1,13) = 43.62, *p* < 0.001, *η*^2^_*p*_ = 0.770]. The lack of vision affected the peak grip aperture as well, with participants exhibiting a significantly larger PGA when no vision was available [*M* = 188.08 mm, *SE* = 5 mm] relative to the full vision condition [*M* = 161.06 mm, *SE* = 4 mm, *F*(1,13) = 125.07, *p* < 0.001, *η*^2^_*p*_ = 0.906]. Relatedly, participants on average achieved their PGA significantly later when no vision was available [*M* = 772 ms, *SE* = 7 ms], as compared to those times when they were allowed full vision during movement [*M* = 695 ms, *SE* = 10 ms, *F*(1,13) = 107.22, *p* < 0.001, *η*^2^_*p*_ = 0.892]. Lastly, as expected, the transport component of the grasp was clearly affected when no visual information was available during the reach-to-grasp movement, with significant decrements recorded for mean peak velocity [*M* = 1.32 m/s, *SE* = 0.6 m/s], mean peak acceleration [*M* = 6.08 m/s, *SE* = 0.04 m/s], and mean peak deceleration [*M* = 4.97 m/s, *SE* = 0.04 m/s], as compared to those transport measures recorded under conditions of full vision [PV: *M* = 1.38 m/s, *SE* = 0.06 m/s, *F*(1,13) = 14.05, *p* = 0.002, *η*^2^_*p*_ = 0.519; PA: *M* = 6.32 m/s, *SE* = 0.04 m/s, *F*(1,13) = 13.44, *p* = 0.003, *η*^2^_*p*_ = 0.508; PD: *M* = 5.35 m/s, *SE* = 0.04 m/s, *F*(1,13) = 8.38, *p* = 0.013, *η*^2^_*p*_ = 0.392].

### Timing by Hand interaction

A significant interaction between the TIMING of tactile stimulation delivery and the HAND executing the movement was found on the total movement time data [*F*(3,39) = 2.91, *p* = 0.047, *η*^2^_*p*_ = 0.183], however, none of the post hoc tests conducted survived the correction for multiple comparisons. The same interaction between the TIMING of tactile stimulation delivery and the HAND executing the movement was also found on the time to peak velocity [*F*(3,39) = 3.62, *p* = 0.021, *η*^2^_*p*_ = 0.218]. Post hoc tests indicated that this result was given by faster time to peak velocity recorded for stimulation delivered in the period before grasp at the moving hand [*M* = 398.88 ms, *SE* = 17 ms], as compared to stimulation delivered to the resting hand [*M* = 411.91 ms, *SE* = 19 ms], *t* (13) = 3.65, *p* = 0.003, *r* = 0.992].

## Discussion

This study investigated the time course of the contribution of visual information to tactile suppression during the execution of a goal-directed reach-to-grasp movement. We focused on the stimulation delivery timings of before grasping an object, as well as when lifting said object, with the purpose of elucidating the specific timing of the previously reported tactile suppression reduction when vision is available^6^. Our participants reached for, grasped, and lifted an object placed centrally on the table in front of them. We expected tactile suppression for the entire time the hand was in motion.

Our results indicate clear tactile suppression for the moving hand, as compared to the resting hand. As expected, tactile suppression magnitude differs among the stimulus delivery timings^3,4,7^, with the worst performance for the moving hand observed the moment before grasping the goal object. A similar pattern was reported for reaches^10^, as well as a significant deterioration in movement accuracy was reported following proprioceptive tendon vibration for the later stages of a goal-directed movement^19^. Even though performance deteriorates at the moving hand for both the preparatory and execution phases of the movement, the recorded average sensitivity is very good and comparable to the previous reports^5,7,11^. This may reflect an almost-ceiling effect given by the utilisation of the 90% detection threshold; future studies need to test a significantly lower threshold (e.g., uniform suppression was described throughout movement for discrimination thresholds tested at 79.4% correct responses^3,20^). Having such a high threshold for detection likely facilitates the “pop-out” of those tactile features known to be easily detected over movement. For example, when participants perform on speeded detection tasks, tactile response times tend to be faster, specifically for the movement execution period, as compared to movement preparation^21^. In a similar fashion, enhanced brain responses have been documented over the execution period of the movement in response to uninformative tactile probes delivered to a moving hand, with the authors suggesting that the processing of incoming tactual information is prioritized with the potential purpose of adjusting the ongoing motor plan, in the eventuality of an unexpected event^22^.

Importantly, suppression was maximal for *the moving hand* specifically at those timings of interest of just before grasping the object and while lifting the object. The availability of visual information clearly influenced participants’ tactile sensitivity: Their sensitivity to detect a tactile stimulus delivered to their moving hand just before grasping the object was significantly higher, when they performed the movement under full vision conditions. A likely contributing factor to this tactile enhancement from vision as found here is the well-demonstrated fact that we reliably tend to fixate near the index finger future contact points on the object^15,16,23^. Additionally, this enhancement effect of what is felt just before grasping an object begs the question regarding what specific type of visual modulation is at play. Specifically, is sensory enhancement at grasp driven by visual attention? Furthermore, is the found sensory enhancement a direct result of the specific type of visual information availability during the reach? If the answer to the latter question is affirmative, then which visual cues contribute to improved tactile sensitivity just before grasping an object: vision of the index finger, or rather, generally vision of the hand and/or object? Recent studies indicate that specific visual information being made available differentially affects the movement profile of the hand^24^. An additional explanation for the enhanced sensitivity found in the full vision condition is that the timing of contact between hand and object could very reliably be predicted when vision is available, as compared to the limited vision condition. This improved temporal prediction could be the trigger for the better tactile detection performance, a result supported by the shorter total movement times recorded when vision was available. That is, vision allows to reliably distinguish the (external) tactile stimulation from any tactile feedback expected/encountered when making contact with the object. The specific visual contribution needs to be ascertained, especially because once the grasp has taken place and the participants are involved in the lift of the object off the table surface, our results further highlight a significantly improved tactile performance at *the resting hand*. For this reason, an additional explanation could be that this visually-triggered enhancement at grasp, and/or lift, is simply the result that the limb is seen, an explanation in line with the classical tactile spatial attention modulations demonstrated in a resting state of the body^25,26^.

It is important to note that our behavioural results demonstrated a clear movement effect on tactual sensation, and this effect was accompanied by a criterion shift. Specifically, participants were more likely to report a lack of tactile stimulation when this was delivered at their moving right hand, as compared to stimulation delivered to their resting left hand. These results are in line with previous reports of a significant conservative criterion shift once a goal-directed movement is initiated^7,11,27,28^. Crucially, the availability of visual information did not affect the relative criterion data, suggesting that the conservative criterion shift is a purely tactually-driven effect, most likely reflecting the perceptual uncertainty given by the ongoing movement (see^1^, for further discussion).

A further point of discussion must acknowledge *how* the vision availability affects the movement profile of the hand. As expected^29–31^, the movement profiles displayed significantly fewer features indicative of closed-loop control when vision was removed. Specifically, movements became longer, with significantly later-occurring and larger peak grip aperture, as well as significant decrements in peak velocity, peak acceleration, and peak deceleration. Moreover, visual cues removal caused significantly faster reaction times to initiate the movement. While this result might seem counterintuitive at first, faster reaction times in the dark likely reflect the exact timing of vision removal, e.g., over the preparatory phase of the movement in the case of our study. Participants are faster to initiate movement so that they reduce the representation of the movement space over time^31,32^. Additionally, supporting the finding underlining that our eyes land at the goal location at the same time as the hand achieving peak acceleration^33^, our results indicate that participants achieve peak velocity faster when tactile stimulation is delivered to the moving hand, as compared to stimulation delivered to the resting hand, specifically just before grasping the goal object.

Taken together, we further confirm the existence of tactile suppression throughout the entire duration of a goal-directed movement. Furthermore, our data indicate that the visual system is at work to counteract this perceptual decrement and act to enhance what is felt at key grasp timings, such that, what we feel at our moving hand is enhanced just before our digits land on the object. Additionally, the resting hand tactile sensitivity seems to also benefit from visual enhancement once the grasp has been resumed and the moving hand is actively making use of the sensory feedback available to perform the lift of the object. Visual availability therefore does not prove beneficial for the lifting phase at the moving hand, but rather seems to be working in favour of enhancing what is felt at the resting hand. This would allow the possibility for our eyes to monitor next points of interest once the object has been grasped and the lifting of it is ongoing. Future studies need to investigate the exact contribution of visual information availability at the moving/resting effectors for differential goals of our actions.

## Methods

### Participants

Twenty participants took part in this study. However, we excluded data from five participants due to technical problems experienced during data collection. The remaining participants comprised 6 male participants, mean age: 26.06 years, SD = 7.76. All participants reported normal or corrected-to-normal vision and no known impairment in their sense of touch. The experiment took approximately 120 minutes to complete and the participants were remunerated 15 EUR for taking part. The study received ethical clearing (CPP SUD EST II) and written informed consent was obtained from all participants before beginning the experiment. All participants were debriefed with respect to the study purpose at the end of experiment. All research was performed in accordance with relevant guidelines (i.e., Public Health Code, Title II of the first book on biomedical research) and regulations (i.e., authorized by AFSSAPS, Agence Française de Sécurité Sanitaire des Produits de Santé – French Agency for Sanitary Security of Health Products). This study conforms to the Declaration of Helsinki and to all subsequent amendments (Declaration of Helsinki, 1964, 2013).

### Apparatus

**Figure 5:**
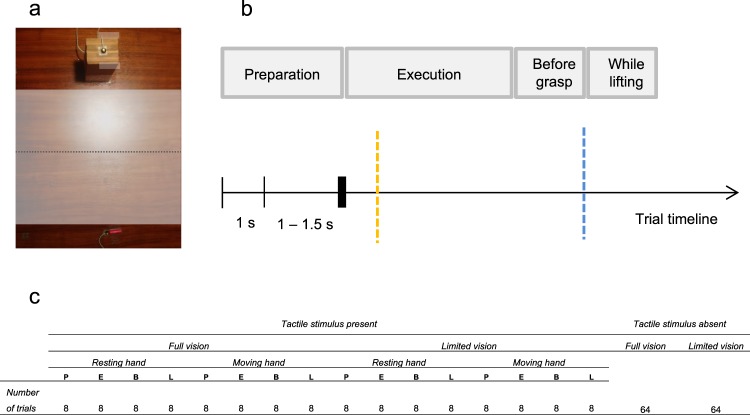
(**a**) Experimental set-up. Participants start each trial by pinch-grasping the start IRED marker. Tactile stimulation could be delivered while preparing the movement, during movement execution (mid-way from start position to goal object, as represented by the dotted line), shortly before the grasp (gray bars indicate spatial landing positions, i.e., 0.5 cm before landing on object, irrespective of elevation, for both index and thumb), and while lifting the object. (**b**) *Trial timeline*. The auditory go signal is depicted with thicker bar, movement initiation in yellow, grasp in blue. (**c**) *Experimental design*. P is Preparation, E is Execution, B is Before grasp, and L is While lifting.

The experiments were conducted in a dark room with illumination provided by a table-top lamp. Participants reached for and grasped a custom-made rectangular object (two-thirds wood and one-third styrofoam, 10 cm tall, 3.8 cm wide, 68 g mass), placed on the table in front of them. See Fig. 5a for a depiction of the object utilized in the study.

Participants wore a pair of liquid crystal display goggles (PLATO goggles, Translucent Technologies, Toronto, ON, Canada) and headphones (ATH-PRO5MK3, Audio-Technica, Tokyo, Japan). Tactile stimulation was delivered by means of two isolated bipolar constant current stimulators (Digitimer DS5, Digitimer Ltd, Welwyn Garden City, UK) which were driven through a NI amplifier (NI USB-6001, National Instruments, Austin, TX, US). Participants had one electrode attached to the ventral part of the fingertip and the ground attached to the middle phalanx of both their index fingers (Neuroline Surface Electrodes 70015-K/12, Ambu AS, Ballerup, Denmark). Movement of participants’ right hand was tracked with an Optotrak Certus (NDI, Waterloo, ON, Canada), positioned at 2.3 meters distance to the left hand side of participants’ start position. Participants wore three infra-red emitting diodes (IREDs) positioned on the index, thumb, and wrist. Extra IRED markers were attached to the table at the start position, to the top of the object, as well as just underneath the object. The experiment was conducted in Matlab (Matlab 2013a, MathWorks, Natick, MA, US), utilizing custom-written scripts in connection to functions from several available toolboxes, such as the Pschychophysics toolbox v3^34,35^, the Optotrack toolbox (V. H. Franz, http://www.ecogsci.cs.uni-tuebingen.de/OptotrakToolbox), and the Data acquisition toolbox.

### Procedure

The experiment consisted of two phases: a thresholding procedure performed at rest and the experimental phase involving goal-directed movements of the right hand.

For the tactile thresholding procedure, we designed two phases. In the first phase, aimed at finding the preliminary detection threshold, the experimenter instructed participants to sit with their eyes closed and both their forearms pronated on the table top. For each hand, we used two intermixed limits staircases^36–38^, with a lower staircase starting at 0 mA (i.e., no stimulation) and a higher one starting at 2.2 mA. That is, there were 4 staircases opened in parallel at the beginning of the procedure. In each trial we delivered a 2 ms square wave pulse stimulus followed, 500 ms later, by an auditory beep (450 Hz, 100 ms) requesting a response from the participants. Participants made a foot-pedal response (stimulus present or absent), irrespective of the hand where this stimulus could be delivered. The inter-trial-interval was set to 2 s. The descending staircases’ step was set at 0.05 mA and the step was doubled for the ascending staircases. Tactile stimulation for the ascending staircases increased one step after each NO response, while it kept the same value following a YES response. Tactile stimulation for the descending staircases decreased one step following a YES response and kept the same value following a NO response. The procedure terminated after four consecutive YES responses for the ascending staircases. These values at the time of termination were taken as the preliminary 90% detection threshold.

In a second phase, to further test the stability of the detection threshold for each hand, we took the preliminary 90% detection threshold values and their corresponding values for the descending staircases at the time of termination and derived 6 more values (by first adding, and then by also subtracting the step value, the doubled step value, or the tripled step value from the detection threshold and the corresponding value from descending staircase, respectively). Altogether we thus computed, for each hand, 8 individual stimulation values. In a separate procedure, for each hand, we administered these 8 values for 10 times, together with 40 trials without stimulation, all randomly intermixed, giving a total of 200 trials/participant. Our particular aim with this extra procedure was to test for false alarms, a procedure which is not available when using the classical adaptive psychophysical measures. At the end of this procedure, the final 90% detection threshold was chosen by the experimenter as the final value of 90% detection stimulation, or, if more available, the highest 90% detection value.

At the beginning of each trial in the experimental phase, participants pinch-grasped the IRED located at the start location (see Fig. 5a). The object was shown for one second. Depending on the trial type (either full vision or limited vision), participants further viewed (or not) the object for a randomly chosen duration between 1 and 1.5 seconds (the randomized foreperiod). This foreperiod was followed by the delivery of the auditory go signal (a beep, 450 Hz, 100 ms). Participants were instructed to reach forward and grasp the object following the delivery of the go signal, shortly lift it off the table, place it back, and return to the start position. They were instructed to only initiate movement upon hearing the go signal and execute an accurate movement at a comfortable speed. Once they returned to the start position, they gave a response with respect to whether they felt the tactile stimulus or not, by means of two foot pedals placed under the table. Response assignments to the left and right pedal (by the ipsilateral foot) were counterbalanced across participants.

The tactile stimulus (a 2 ms square wave, its amplitude established during thresholding procedure) could be delivered at four different timings: *(1)* during *movement preparation* (following the initial one second period where we showed the object, the beep was played halfway into the randomized foreperiod used); *(2)* during movement execution (delivered once the hand travelled more than 15 cm from the start position, that is, half of the total distance); *(3)* just before fingers contacted the object (when the hand was still in motion and both the index and the thumb were detected within less than 0.5 cm from landing on the object); *(4)* while lifting the object (when the hand was in motion lifting the object, and the IRED marker positioned underneath the object became visible). See Fig. 5b for a depiction of the trial timeline.

### Design

The experimental phase consisted of 4 blocks of 64 trials each, with a total of 256 trials. Half of the trials were stimulus present trials, whereas in the other half no stimulation was delivered. Half of the total number of trials were conducted under full vision for the entire duration of the trial, whereas for the remaining half participants were given only 1 second of visual information at the beginning of the trial, with the reach-and-grasp movement being performed with closed goggles. Further, if tactile stimulation was present, in half of the trials tactile stimulation was delivered at the resting left hand, and the other half at the moving right hand. Lastly, for each type of vision availability, for each hand, stimulation could be delivered during either the motor preparation period, during execution, just before the grasp, or while lifting the goal object. See Fig. 5c for experimental design.

### Data collection and reduction

The six IREDs data were sampled at 250 Hz for a total time of 4 s. For each trial, the displacement data were filtered offline with a second order dual-pass Butterworth filter, employing a low-pass cut-off frequency of 10 Hz. The analysis program derived velocities by differentiating the displacement data with a three-point central finite difference algorithm. The analysis program further differentiated displacement data to obtain acceleration. The kinematic analysis program defined movement initiation by determining the first sample after which the velocity of the IRED attached to participants’ wrist attained and maintained a value of 50 mm/s for ten consecutive frames (i.e., 50 ms). Contrastingly, movement offset was defined as the point at which the wrist IRED fell below 50 mm/s and remained below this criterion for ten consecutive frames (i.e., 50 ms). If visibility of any of the three IREDs attached to the participants’ hand was lost for the duration of the trial, the trial was repeated at the end of the experiment.

### Statistical analysis

Statistical analysis was performed on both behavioural response data and the kinematic movement data recorded. The data that support the findings of this study are available from the corresponding author, [GJ], upon request.

### Behavioural data analysis

For each participant and for each of the conditions (see Fig. 5c), hit rate (i.e., YES responses when a tactile stimulus was delivered), as well as false alarms (i.e., YES responses when no tactile stimulus was present) were calculated. Experimental conditions were split considering the manipulated experimental variables of TIMING of stimulation (preparation versus execution versus before grasp versus while lifting), VISION AVAILABILITY (full vision versus limited vision), and HAND receiving the stimulation (resting hand versus moving hand). These percentages were normalized and sensitivity measures (*d’*) and the relative criterion *c’* were derived according to signal detection theory (SDT^13,14^, see also^27,28^ for similar methods). Whenever accuracy was perfect for a given condition (i.e., participants always detected the tactile stimulus), or no false alarms were recorded, the proportions of 1 and 0 were adjusted by 1/(2 *N*), and 1/(1–2 *N*), respectively, where *N* is the number of trials for a given condition on which the proportion was calculated^39^.

For each of the derived SDT measures (*d’* and *c’*) we performed repeated-measures analyses of variance (ANOVAs) with the factors TIMING of tactile stimulation (preparation versus execution versus before grasp versus while lifting), VISION AVAILABILITY (full vision versus limited vision), and HAND receiving the stimulation (resting hand versus moving hand). Mauchly’s test of sphericity was used to identify violations of the sphericity assumption. If the assumption was violated, then the Greenhouse-Geisser correction was applied to correct the degrees of freedom; corrected *p* values are reported throughout. Hypothesis-driven analyses of variance followed any three-way interaction found in the data. Sidak-corrected paired-samples *t*-tests followed two-way interactions found in the data. Partial *η*^*2*^ is reported as an effect size estimate for the ANOVA results; the correlation coefficient *r* is used as effect size for the *t*-tests. For all the analyses, only those significant main effects and interactions found in the data are reported.

### Kinematic data analysis

The kinematic dependent measures considered were: reaction time (RT), total movement time (MT), peak grip aperture (PGA), peak velocity (PV), peak acceleration (PA), and peak deceleration (PD), together with their latencies, that is the time needed to reach each of the PGA, PV, PA, and PD. For each of these kinematic measures separate repeated-measures ANOVAs were conducted with the same factors as for the behavioural statistical analysis. One participant was excluded from the kinematic analysis as we consistently missed the IRED markers during movement.
